# Gender Differences in Generating Cognitive Reappraisals for Threatening Situations: Reappraisal Capacity Shields Against Depressive Symptoms in Men, but Not Women

**DOI:** 10.3389/fpsyg.2019.00553

**Published:** 2019-03-15

**Authors:** Corinna M. Perchtold, Ilona Papousek, Andreas Fink, Hannelore Weber, Christian Rominger, Elisabeth M. Weiss

**Affiliations:** ^1^Department of Psychology, University of Graz, Graz, Austria; ^2^Department of Psychology, University of Greifswald, Greifswald, Germany

**Keywords:** cognitive reappraisal, emotion regulation, gender differences, depression, maximum performance

## Abstract

Despite major research interest regarding gender differences in emotion regulation, it is still not clear whether men and women differ in their basic capacity to implement specific emotion regulation strategies, as opposed to indications of the habitual use of these strategies in self-reports. Similarly, little is known on how such basic capacities relate to indices of well-being in both sexes. This study took a novel approach by investigating gender differences in the capacity for generating cognitive reappraisals in adverse situations in a sample of 67 female and 59 male students, using a maximum performance test of the inventiveness in generating reappraisals. Participants’ self-perceived efficacy in emotion regulation was additionally assessed. Analyses showed that men and women did not differ in their basic capacity to generate alternative appraisals for anxiety-eliciting scenarios, suggesting similar functional cognitive mechanisms in the implementation of this strategy. Yet, higher cognitive reappraisal capacity predicted fewer depressive daily-life experiences in men only. These findings suggest that in the case of cognitive reappraisal, benefits for well-being in women might depend on a more complex combination of basic ability, habits, and efficacy-beliefs, along with the use of other emotion regulation strategies. The results of this study may have useful implications for psychotherapy research and practice.

## Introduction

Among the most pervasive differences between men and women in the realm of emotion is women’s heightened vulnerability toward the development of affective disorders, in particular depression and anxiety (e.g., Nolen-Hoeksema, 2001; Kessler et al., 2007; Steel et al., 2014). Over the years, this female proneness to depressive symptoms has been attributed to heightened emotional reactivity toward negative stimuli (Bradley et al., 2001; Kessler, 2003; Kelly et al., 2008) as well as potentially maladaptive emotion regulation (e.g., Garnefski et al., 2004; Nolen-Hoeksema, 2012), both behaviorally and on the level of the brain (e.g., Domes et al., 2010; Whittle et al., 2011; Stevens and Hamann, 2012). However, consistent empirical support for sex differences especially in emotion regulation that may in turn elucidate gender differences in several types of psychopathology is sparse (see Nolen-Hoeksema and Aldao, 2011; Whittle et al., 2011; Zimmermann and Iwanski, 2014). This, along with increasing recognition that deficient emotion regulation is at the core of various disorders (Martin and Dahlen, 2005; Aldao and Nolen-Hoeksema, 2010; Hofmann et al., 2012; Berking et al., 2014; Joormann and Stanton, 2016), highlights the need for more in-depth investigations on gender differences^1^ in the proficiency of implementing certain emotion regulation strategies.

One emotion regulation strategy that merits special attention in this case is cognitive reappraisal. Cognitive reappraisal aims at changing the emotional impact of a situation by deliberately viewing it from a different perspective by using alternative situational interpretations (e.g., Lazarus and Alfert, 1964; Lazarus and Folkman, 1984; Gross and John, 2003). Converging evidence from multiple studies has shown that cognitive reappraisal is particularly powerful in dealing with adverse events, sustainably regulating negative affect and decreasing depressive symptoms (e.g., Martin and Dahlen, 2005; Augustine and Hemenover, 2009; Troy et al., 2010; Webb et al., 2012). In this respect, Martin and Dahlen (2005) found that independent of gender, higher self-reported positive reappraisal predicted lower depressive symptoms, while Troy et al. (2010) showed that cognitive reappraisal protected against depressive symptoms during stressful life events. Meta-analyses corroborated these findings, with Augustine and Hemenover (2009) demonstrating links between cognitive reappraisal and large hedonic shifts in affect (defined as decreases in negative or increases in positive emotions and indexed by self-report). These findings were supported by the meta-analysis of Webb et al. (2012), who reported cognitive reappraisal to be highly effective in modifying emotional outcomes on behavioral and physiological levels as well. While this invites assumptions that a higher prevalence of depression in women may partly originate from less frequent or less effective use of cognitive reappraisal, available data are mixed. According to some studies, women employ cognitive reappraisal on a more frequent basis than men do (e.g., Tamres et al., 2002; Spaapen et al., 2014; also see Nolen-Hoeksema, 2012), though in the meta-analysis of Tamres et al. (2002), this effect was reported for most emotion regulation strategies. These findings are, however, challenged by others that report no gender differences in the habitual use of cognitive reappraisal (Gross and John, 2003; Haga et al., 2009; Zlomke and Hahn, 2010), or even endorse more positive re-interpretations in men (Öngen, 2010). Research on gender-specific effects of cognitive reappraisal use on depressive symptoms during adolescence yielded disparate results as well, either denoting cognitive reappraisal equally effective in attenuating depressive symptoms in both men and women (Shapero et al., 2018) or suggesting that greater habitual use of cognitive reappraisal more strongly decreases depressive symptoms in adolescent girls than boys (Duarte et al., 2015).

One possible explanation for these inconclusive results is that, while having provided vital evidence, these approaches mainly focused on self-reported tendencies to use cognitive reappraisal, thereby neglecting potential gender differences in actual capacity to adequately implement cognitive reappraisals in critical situations (e.g., Perchtold et al., 2018). Several researchers pointed out that individuals’ typical reappraisal use in daily life cannot be equated with their actual capacity to use this strategy when confronted with adverse scenarios, given the absence of or only weak correlations between the two (McRae et al., 2008; Troy et al., 2010; Weber et al., 2014). However, despite numerous appeals for more objective performance measures of individuals’ actual emotion regulation capacity (Demaree et al., 2006; McRae et al., 2008; Whittle et al., 2011; Opitz et al., 2015), few efforts have been made in that direction. Thus, assumptions that men and women may differ in their basic capacity for cognitive reappraisal remain rather speculative to date. In an attempt to add some clarity to the picture, two brain imaging studies (McRae et al., 2008; Domes et al., 2010) specifically investigated sex differences in neural correlates of instructed cognitive reappraisal, albeit with different outcomes. McRae et al. (2008) reported lower increases in prefrontal activity and greater decreases in amygdala activity during reappraisal efforts in men compared to women, despite similar attenuations of self-reported negative emotions in both sexes. Domes et al. (2010) found quite the opposite activation pattern, indicating greater prefrontal activity in men compared to women during cognitive reappraisal implementation, with no notable sex differences in amygdala activity or self-report regulation success. Intriguingly, both studies interpreted their results in terms of a more efficient reappraisal process in men, suggesting less effortful cognitive control (McRae et al., 2008) and more appropriate recruiting of regulatory areas (Domes et al., 2010) in men compared to women. Although this argument critically implicates executive control processes in effective reappraisal (Joormann and Gotlib, 2010; Malooly et al., 2013; Pe et al., 2013; Rominger et al., 2018), neither study used objective behavioral indicators of reappraisal capacity, making it difficult to put their findings into perspective. Altogether, the question whether men and women differ in their basic capability for implementing alternative appraisals in critical situations is thus still unanswered.

The present study aims to address this gap in literature by investigating gender differences in the basic capacity for generating cognitive reappraisals. Moreover, it was examined how this capacity relates to individuals’ depressive daily-life experiences. More precisely, we sought to determine whether cognitive reappraisal capacity may serve as a predictor of depressive experiences in daily life also over and above individuals’ self-efficacy in the regulation of emotions, and whether this holds for both genders in a similar way. In this study, we used the Reappraisal Inventiveness Test (RIT; Weber et al., 2014), which confronts individuals with self-relevant, threatening situations and instructs them to produce as many different cognitive reinterpretations as possible in order to downregulate their experienced stress and anxiety. Importantly, by using the RIT, our focus was on gender differences in reappraisal capacity in the psychometric sense, that is, to what degree men and women are theoretically capable of implementing cognitive reappraisal in aversive situations (maximum performance, Cronbach, 1970). Objective coding of participants’ reappraisal ideas in terms of appropriateness (see Demaree et al., 2006) then results in an index of reappraisal capacity. This capacity can be referred to as basic or fundamental, as it delineates an individuals’ basic cognitive potential to construct different interpretations for given situations in the first place (i.e., a construction competence), allowing for more flexibility in coping with everyday challenges (Weber et al., 2014). In this regard, studies have linked higher cognitive reappraisal capacity to more appropriate recruitment of the lateral prefrontal cortex during emotion regulation efforts (Papousek et al., 2017), which also predicted self-perceived chronic stress levels (Perchtold et al., 2018). This corroborates the notion that this brain-based cognitive reappraisal capacity may affect more distal emotional outcomes like stress perception and by implication, possibly depressive experiences. Thus, cognitive reappraisal capacity likely constitutes a necessary prerequisite for effective reappraisal implementation in daily life (Weber et al., 2014; de Assuncao et al., 2015; Papousek et al., 2017). However, in this regard, two things need to be considered. Firstly, in daily life, it might occasionally seem more relevant to produce one high-quality reappraisal than a variety of different reappraisals to effectively diminish the emotional impact of aversive situations. Yet, it can be argued that the capacity to generate a large pool of potential reappraisals for a given situation makes it more likely to select reappraisals individuals can effectively implement in this specific context (also see Wisco and Nolen-Hoeksema, 2010). Having a broad repertoire of potential reappraisals readily available may be especially relevant when individuals face new situations, in which they cannot rely upon their routine strategies (Weber et al., 2014). Secondly, though considered a vital prerequisite for effective cognitive reappraisal implementation, reappraisals capacity only covers a certain aspect in the reappraisal process, as individuals not only need to be principally capable of constructing various situational appraisals, they also need to make use of this ability in daily life (Perchtold et al., 2018). Conversely, however, if individuals’ basic capacity for cognitive reappraisal generation is impaired, habitual use of cognitive reappraisal in daily life may not yield any benefits, and reappraisal trainings, e.g., in cognitive behavioral therapy, may not be sufficiently effective.

To the best of our knowledge, no study to date has tested gender differences in the explicit ability to *ad hoc* generate cognitive reappraisals for adverse situations. Moreover, given equivocal evidence from literature as to sex differences in executive control processes relevant to emotion regulation (McRae et al., 2008; Domes et al., 2010; Franklin et al., 2018), we did not have strong *a priori* predictions regarding which gender would show better cognitive reappraisal capacity and how this capacity would relate to depressive symptoms in men and women. In line with available literature, however, we did hypothesize that women would report more depressive experiences than men (Nolen-Hoeksema, 2001; Van de Velde et al., 2010; Salk et al., 2017) and conversely, less self-efficacy in emotion regulation (e.g., Freudenthaler and Papousek, 2013). A relationship between cognitive reappraisal capacity and self-efficacy beliefs seems likely, with self-efficacy potentially acting as the decisive variable for daily-life experience of depression. In this regard, previous research reported substantial correlations between perceived self-efficacy in emotion regulation and various indexes of well-being (see Baudry et al., 2018). Additionally, in light of recent findings that some cognitive reappraisal strategies (e.g., positive re-interpretations) might be more adaptive than others as regards implications for well-being (Kalisch et al., 2015; Willroth and Hilimire, 2016; Perchtold et al., 2018), we tested for gender differences in the quality of generated reappraisals (positive re-interpretation, de-emphasizing, problem-orientation, symptom re-interpretation).

## Materials and Methods

### Participants

The sample comprised 126 participants (67 women, 59 men), aged between 18 and 35 (*M* = 22.42, *SD* = 3.15). All participants were university students enrolled in various fields. No participant reported using drugs or psychoactive medication and none had participated in an experiment using the RIT before. Thirty women reported the use of hormonal contraceptives, with *n* = 25 using the contraceptive pill (duration of use: *M* = 3.86 years *SD* = 2.59), and *n* = 5 using intrauterine devices (duration of use: *M* = 2.04 years; *SD* = 1.16). The study was approved by the authorized ethics committee. Participants gave their written consent to participate in the study. After receiving general instructions, participants completed the RIT and questionnaires.

### Reappraisal Inventiveness Test (RIT)

The RIT (Weber et al., 2014) is a maximum performance test for cognitive reappraisal ability that confronts individuals with adverse emotional situations likely to occur in their everyday lives. Participants are instructed to imagine the situation happening to them and to generate and write down as many different ways as possible to think about the situation in a way that diminishes their negative emotions. In the present study, four vignettes depicting anxiety-eliciting situations (de Assuncao et al., 2015) were presented one at a time on separate pages and were supplemented by a picture in order to make them more vivid. For each vignette, participants were given 20 s to imagine the situation happening to them and then turn to the next page at the signal of the experimenter. Subsequently, participants wrote down as many different ways to reappraise the situation with the goal to diminish anxiety until the allotted time of 3 min per situation had elapsed. In the night item of the RIT (situation 1), for instance, participants face the following situation: “*At night, you lie alone in bed and are about to fall asleep, when you suddenly hear a loud noise from the living room. You get up, go into the living room and realize that the window is open.”* In the other situations, individuals are confronted with walking home alone at night (2), a root canal appointment (3), and a smoke alarm going off at the neighbors (4). For the assessment of behavioral measures of their reappraisal inventiveness, participants’ responses to the RIT items were used and independently rated by two experienced experimenters, who received extensive training beforehand.

### Cognitive Reappraisal Capacity

Following the scoring procedure of the RIT and previous relevant research (Weber et al., 2014; Fink et al., 2017; Papousek et al., 2017; Perchtold et al., 2018; Rominger et al., 2018), RIT-fluency was used as an index of cognitive reappraisal capacity, calculated as the total number of generated non-identical reappraisals (α = 0.93). On average, participants generated *M* = 22.12 (*SD* = 5.23) valid reappraisals. The number of reappraisal ideas generated for each of the four situations differed slightly, with significantly fewer ideas generated for situation 4 (*M* = 5.12) than for the rest (situation 1: *M* = 5.75, *p* < 0.001; situation 2: *M* = 5.74, *p* < 0.001; situation 3: *M* = 5.51, *p* = 0.060). The inter-rater reliability with two-way random, single measure ICC (95% confidence intervals, consistency) was = 0.99 for overall RIT-fluency. Reappraisal were additionally categorized according to the category scheme of the RIT (Weber et al., 2014), which allows for a more profound categorization of reappraisal ideas according to content. The four reappraisal categories in the RIT are: positive re-interpretation (generating positive aspects; *M* = 8.64, *SD* = 4.46; e.g., “Now that I am awake, I get to do some stargazing”), de-emphasizing (trivializing the impact of the situation; *M* = 9.54, *SD* = 4.03; e.g., “Why would someone break into my apartment, I do not own anything valuable”), problem-orientation (finding ways to reduce harm; *M* = 3.27, *SD* = 3.47; “I have my phone, I can call for help anytime”), and symptom re-interpretation (reappraising physical arousal; *M* = 0.35, *SD* = 0.62; e.g., “My heart is just beating rapidly because I got out of bed so fast”). For more example answers matched to their respective category, please see Supplementary Appendix. Other reappraisal ideas not matching these four categories were excluded due to lack of respective answers generated by the participants. Inter-rater reliabilities were ICC = 0.96, ICC = 0.95, ICC = 0.97, and ICC = 0.89 for positive re-interpretation, de-emphasizing, problem-orientation, and symptom re-interpretation, respectively. After completion of all vignettes, participants rated the extent of anxiety they would experience when confronted with the depicted situations (7-point scales ranging from 0 “not anxious at all” to 6 “very anxious”). Ratings were *M* = 3.56 (*SD* = 1.78), *M* = 3.40 (*SD* = 1.68), *M* = 2.72 (*SD* = 1.78), and *M* = 3.18 (*SD* = 1.47). In one-sample *t*-tests, ratings for all vignettes differed significantly from zero (*t*-values ranging from 17.14 to 24.27, all *p*-values <0.001), indicating that all situations were indeed perceived as anxiety evoking. Situation 3 (*M* = 2.72) was perceived as significantly less anxiety evoking than situation 1 (*p* = 0.003) and situation 2 (*p* = 0.017).

### Self-Report Measures

#### Depression

The Center for Epidemiologic Studies Depression Scale (CES-D, German version; Hautzinger and Bailer, 1993) is comprised of 20 items, rated from 0 (rarely or none of the time – less than 1 day) to 4 (most or all the time – 5 to 7 days; α = 0.90). It refers to mood and attributions over the past week and is designed for measuring sub-clinical depressive daily-life experiences in the general population (Wood et al., 2010). Scores ranged from 0 to 37 (*M* = 12.05, *SD* = 7.0).

#### Perceived Efficacy in Managing Negative Emotions

The emotion regulation subscale of the Self-report Emotional Ability Scale (SEAS; Freudenthaler and Neubauer, 2005) was used to assess how able individuals feel to regulate negative affect in their everyday life (e.g., “It is easy for me to change my bad mood”). The 6 items are rated on 6-point Likert scales ranging from 1 to 6 (α = 0.75). Scores ranged from 9 to 34 (*M* = 22.51, *SD* = 5.17).

### Statistical Analysis

In order to investigate basic gender differences in the central variables of interest (cognitive reappraisal capacity, self-efficacy in managing negative emotions), two independent sample *t*-tests were computed. Subsequently, a three-step hierarchical multiple regression analysis was employed with depression as the dependent variable. In the first step, gender was entered as a predictor of depressive daily-life experiences. The second step added reappraisal capacity and self-efficacy in emotion regulation as predictors, with the third step additionally considering interactions of gender and reappraisal capacity, as well as of gender and perceived self-efficacy in managing negative emotions. The applied hierarchical regression approach allowed to examine, firstly, whether men and women differ in the amount of depressive experiences in their everyday lives (first step). Secondly, it examined whether gender differences in depressive experiences are explained by individual differences in reappraisal capacity and/or self-efficacy in managing negative emotions, and whether these variables as such are related to depression (i.e., explain unique variance in the amount of depressive experiences beyond that afforded by gender differences; second step). Thirdly, it allowed to examine whether potential relationships between reappraisal capacity and self-efficacy in managing negative emotions with depression are differently expressed for men and women (third step of the hierarchical regression). The statistical assumptions for the model (i.e., ratio of cases to independent variables, normality, independence of errors, homoscedasticity, linearity, and absence of multicollinearity) were met. A significance level of *p* < 0.05 (two-tailed) was used. Additionally, a multivariate analysis of variance was computed to test for potential gender differences in the patterns of used reappraisal categories (number of reappraisals qualifying as positive re-interpretation, de-emphasizing, problem-orientation, and symptom re-interpretation).

## Results

### Basic Gender Differences in Cognitive Reappraisal Capacity and Self-Efficacy in Managing Negative Emotions

In terms of perceived self-efficacy in managing negative emotions, men reported significantly higher self-efficacy than women [men: *M* = 24.46, *SD* = 4.93; women: *M* = 20.79, *SD* = 4.77; *t*(124) = 4.24, *p* < 0.001]. However, men and women did not differ in their basic capacity to generate cognitive reappraisals for anxiety-eliciting events [men: *M* = 5.55, *SD* = 1.24; women: *M* = 5.52, *SD* = 1.37; *t*(124) = 0.118, *p* = 0.906]. Moreover, while women reported feeling greater anxiety elicited by the presented scenarios [men: *M* = 2.81, *SD* = 0.12; women: *M* = 3.58, *SD* = 0.69; *t*(124) = -5.27; *p* < 0.001], this self-reported anxiety was uncorrelated with performance on the reappraisal test (*r* = -0.07, *p* = 0.468). No significant differences in any variables of interest were observed between women who did and those who did not report using hormonal contraceptives (all *p*’s > 0.140).

### Relationships Between Cognitive Reappraisal Capacity and Self-Efficacy in Managing Negative Emotions With Depressive Experiences in Men and Women

**FIGURE 1:**
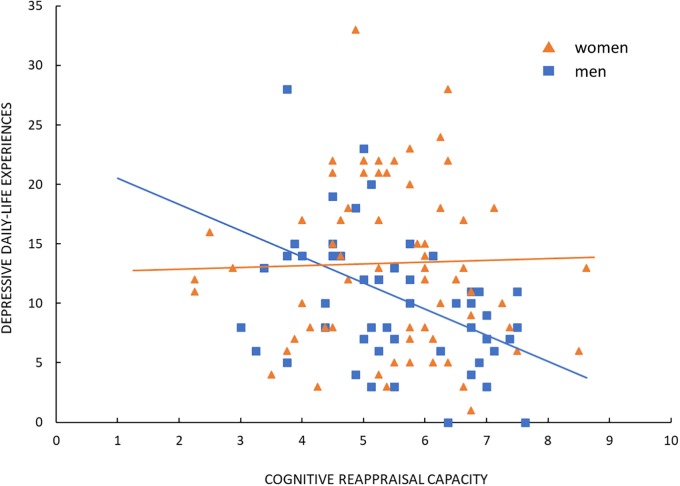
Interaction effect of cognitive reappraisal capacity by gender on depressive daily-life experiences.

In Table 1, the findings of the hierarchical regression analysis are summarized. At step one, gender significantly correlated with the amount of depressive experiences [*r* = 0.21; *F*(1,124) = 5.57, *p* = 0.020], indicating that, overall, women reported more depressive experiences than men (men: *M* = 10.51, *SD* = 6.35; women: *M* = 13.40, *SD* = 7.30). In addition to gender, reappraisal capacity and self-efficacy explained additional 21% of the variance in depressive experiences [*F*(3,122) = 13.67, *p* < 0.001]. While both of these variables explained unique portions of variance in depression (reappraisal capacity: *sr* = -0.18, *p* = 0.027; self-efficacy: *sr* = -0.43, *p* < 0.001), the contribution of gender became non-significant (*sr* = 0.04, *p* = 0.621) as reappraisal capacity and self-efficacy were included in the model. Together, this suggests that the observed gender differences in reported depressive experiences are to a large part attributed to differences in self-efficacy in emotion regulation. Overall, higher scores in self-efficacy as well as in cognitive reappraisal capacity were associated with less depressive experiences. Entering the interaction terms reappraisal capacity by gender and self-efficacy by gender in the model additionally increased the explained amount of variance in the experience of depression by 4% [*F*(5,120) = 9.82, *p* < 0.001]. Of the two interactions, only the contribution of the interaction reappraisal capacity by gender was significant (*sr* = 0.18, *p* = 0.020; self-efficacy by gender: *sr* = -0.07, *p* = 0.357). The significant interaction indicates that while a higher basic capacity for cognitive reappraisal generation for anxiety-eliciting situations was associated with lower self-reported depressive experiences in men, the capacity for reappraisal generation was unrelated to the experience of depression in women (men: *r* = -0.42, *p* < 0.001; women: *r* = 0.03, *p* = 0.820). See Figure 1 for an illustration of the significant interaction effect.

**Table 1:** Summary of hierarchical multiple regression results.

	*β*	*p*	Δ*R*^2^	*p*
Step 1				
Gender	0.207	0.020		
			0.043	0.020
Step 2				
Gender	0.042	0.621		
Cognitive reappraisal capacity	-0.175	0.027		
Self-efficacy in emotion regulation	-0.460	<0.001		
			0.209	<0.001
Step 3				
Gender	0.049	0.552		
Cognitive reappraisal capacity	-0.211	0.008		
Self-efficacy in emotion regulation	-0.442	<0.001		
Reappraisal capacity × gender	0.184	0.020		
Self-efficacy × gender	-0.071	0.357		
			0.039	0.042


*Dependent variable: amount of depressive daily-life experiences (CES-D). Gender was scored such that the positive beta weight indicates that women reported more depressive experiences than men. For an illustration of the significant interaction effect see Figure 1. Full model: *F*(5,120) = 9.82, *p* < 0.001*.

In light of evidence that the difficulty of cognitive reappraisal increases with the intensity of emotional situations (e.g., Sheppes et al., 2014), we additionally ran two separate hierarchical regression analyses for the lower and higher anxiety eliciting items. In both analyses, the previously observed interaction reappraisal capacity by gender remained significant (lower anxiety eliciting: *sr* = 0.19, *p* = 0.019; higher anxiety eliciting: *sr* = 0.16; *p* = 0.042), indicating that differences in anxiety ratings for the RIT vignettes did not influence the main findings of this study.

### Gender Differences in Use of Reappraisal Sub-Strategies

Men and women did not differ in their employment of different reappraisal strategies [*F*(4,121) = 1.04, *p* = 0.387]. See Table 2 for a descriptive summary of the rates of generated reappraisal categories. On an exploratory basis, it was additionally examined how the use of different reappraisal strategies contributed most to the reporting of depressive daily-life experiences (standard multiple regression analysis). The generation of relatively more reappraisals categorized as de-emphasizing (*sr* = -0.16, *p* = 0.038) and positive re-interpretation (*sr* = -0.15, *p* = 0.064) were associated with fewer depressive experiences, whereas the use of problem orientation (*sr* = 0.03, *p* = 0.724) and symptom re-interpretation (*sr* = -0.10, *p* = 0.214) did not seem to play an important role on their own [*F*(6,119) = 7.68, *p* < 0.001]. This result was independent from variance explained by gender and self-efficacy in managing negative emotions.

**Table 2:** Use of reappraisal strategies, expressed as percentage of total generated cognitive reappraisals.

	Positive re-interpretation	De-emphasizing	Problem-orientation	Symptom re-interpretation
Women	38.29	41.48	17.34	0.98
Men	38.56	45.65	12.60	1.37


## Discussion

This study examined gender differences in the fundamental capacity to spontaneously generate alternative cognitive reappraisals for anxiety-eliciting scenarios as well as their potential relevance to depressive experiences in everyday life. In line with indications of greater emotional reactivity to negative information and stressful events in women than men as well as women’s greater proneness to clinical depression (Bradley et al., 2001; Nolen-Hoeksema, 2001; Kessler et al., 2007; Kelly et al., 2008; Steel et al., 2014), women reported more depressive symptoms than men in the current study. Nevertheless, these differences were not reflected in basic reappraisal skills, as men and women demonstrated a similar capacity to generate meaningful alternative interpretations for adverse anxious events. This constitutes a novel finding in literature, as potential gender differences in emotion regulation capacity have never been scrutinized with a maximum performance test of reappraisal ability before. Despite previous studies hinting at a more efficient reappraisal process in men based on their prefrontal cortex engagement and related stronger executive functioning (e.g., McRae et al., 2008; Domes et al., 2010; also see Masumoto et al., 2016), this study yielded no evidence suggesting a potential advantage of men in the behavioral test for reappraisal inventiveness. Note that while greater reappraisal inventiveness does not automatically translate to efficacy in cognitive reappraisal, it may inform about vital cognitive prerequisites of efficient reappraisal implementation. Accordingly, based on their performance in this study, men and women presumably recruit similar functional executive processes during reappraisal generation, of which set-shifting, memory updating, and inhibition of dominant yet irrelevant responses are proposed as crucial building blocks for cognitive reappraisal (Joormann and Gotlib, 2010; Malooly et al., 2013; Pe et al., 2013). Since the importance of executive functions has also been endorsed by specific research on reappraisal inventiveness (Weber et al., 2014; Papousek et al., 2017; Perchtold et al., 2018; Rominger et al., 2018), our findings suggest equivalent executive functioning in both genders as regards cognitive reappraisal.

Interestingly, however, a higher capacity for reappraisal generation predicted fewer depressive symptoms in men only, while this effect was absent in women. Hence, our results indicate that while both genders do not differ in their basic reappraisal capacity, this capacity appears to be a protective buffer against depression in men only. Although it is premature to draw any firm conclusions from this novel observation, the trends in this study prompt us to speculate on some non-competing explanations for this result. A possible explanation for the observed null effects of reappraisal capacity on depression in women could be linked with the finding of lower self-efficacy in managing negative emotions in women than in men. Substantial positive effects of emotion regulation self-efficacy on well-being are abundant in literature (see Baudry et al., 2018 for review). Further, it was suggested that individuals with higher self-efficacy in emotion regulation put more efforts in actively modifying their emotions and, hence, are prone to use effortful regulation strategies such as cognitive reappraisal more consistently (Tamir et al., 2007). On that note, findings showed that individuals regarding themselves more capable of controlling their emotions were more prone to use cognitive reappraisal in their daily lives. Furthermore, those individuals who more persistently used cognitive reappraisal and scored higher on emotion regulation self-efficacy were more successful in downregulating negative emotions (Gutentag et al., 2017).

Thus, for the present study, the following tentative interpretation is suggested: Men, due to higher confidence in their emotion regulation skills, could generally show greater attempts to actively cope with adverse events, and use effortful active regulation strategies such as cognitive reappraisal with greater determination than women do. Thereby, they may benefit from good reappraisal capacity in terms of fewer depressive daily-life experiences. In contrast, good reappraisal capacity might be less significant for the experience of depression in women because based on lower self-perceived regulation skills, they show reduced emotion regulation attempts from the start. It is thus assumed that effort or motivation in using cognitive reappraisal may be more important than a more frequent employment of cognitive reappraisal alone, as suggested by several indications that men tend to report less habitual use of reappraisal than women (Tamres et al., 2002; Nolen-Hoeksema and Aldao, 2011; Spaapen et al., 2014), although this assumption is not corroborated by all studies (Gross and John, 2003; Haga et al., 2009; Zlomke and Hahn, 2010). Specifically for anxiety-eliciting situations, it is possible that women are less motivated to downregulate anxiety by means of cognitive reappraisal, since they are more prone to feelings of anxiety (e.g., McLean and Anderson, 2009) and are thus more likely to accept these feelings as part of their everyday lives. Complementing this assumption, despite good reappraisal capacity, women might also be less convinced of the effectivity of cognitive reappraisal in reducing their anxious feelings, which adds beliefs about consequences of cognitive reappraisal as another potential influencing factor (e.g., Ortner et al., 2017). Our data, however, can only partly support all these arguments, because we did not assess efforts put in the reappraisal task, beliefs in reappraisal effectiveness, and the preferred use of cognitive reappraisal as a trait (e.g., Gross and John, 2003).

Additionally, it can be derived from literature that women tend to report using both, adaptive and maladaptive emotion regulation strategies more than men (Thoits, 1991; Tamres et al., 2002; Nolen-Hoeksema and Aldao, 2011). While this at first underlines a supposedly more flexible repertoire of regulation strategies in women, there are also studies suggesting that maladaptive emotion regulation strategies (e.g., rumination, suppression) are more strongly linked to depression than are adaptive ones (e.g., cognitive reappraisal, acceptance; Aldao et al., 2010; Nolen-Hoeksema and Aldao, 2011; Joormann and Stanton, 2016). As a consequence, if women endorse more maladaptive regulation strategies than men, and if these strategies were eminently detrimental to mental well-being (e.g., Nolen-Hoeksema et al., 2008, also see Krause et al., 2017), good cognitive reappraisal capacity alone may not suffice to guard against the experience of depression in women, as the impact of concomitantly employed maladaptive strategies prevails. It is hence possible that in women, interactions between adaptive and maladaptive emotion regulation strategies have a more pronounced impact on depressive experiences than the capability to effectively implement one adaptive strategy *per se*.

In line with recent indications that some reappraisal strategies might be more adaptive than others in the long run (Kalisch et al., 2015; Perchtold et al., 2018, 2019), this study also examined gender differences in four reappraisal categories scored in the cognitive reappraisal test (Weber et al., 2014; de Assuncao et al., 2015). No differences emerged, however, despite some evidence that men more often employ problem-oriented coping strategies (Ptacek et al., 1994; Baker and Berenbaum, 2007), whereas women favor emotion-focused tactics (Lazarus and Folkman, 1984; Eaton and Bradley, 2008). It appears that these allegedly basic preferences are not reflected in reappraisal categories. Yet, further research is warranted to look more closely into potential gender differences among the myriad of available strategies that occur in cognitive reappraisal of aversive events (e.g., McRae et al., 2012; Perchtold et al., 2019). Independent of gender and other strategies, the generation of relatively more de-emphasizing reappraisals and positive re-interpretations was associated with fewer depressive experiences. This result supports previous studies that find both, self-focused (de-emphasizing) and situation-focused (positive) reappraisal effective in reducing negative emotional reactivity (Shiota and Levenson, 2012; Ranney et al., 2017), albeit more long term-benefits are suggested for positive reappraisal (e.g., Kalisch et al., 2015).

Importantly, in the present study, the obtained differences in reappraisal capacity effects on depressive experiences between men and women cannot be definitively interpreted in terms of sex or gender. Cognitive reappraisal capacity reflects individuals’ capability to recruit appropriate brain activation when faced with the demand of reappraising an aversive event (Papousek et al., 2017; Perchtold et al., 2018). Since no differences in this basic capacity were observed, this potentially also points to the absence of sex differences in recruitment of adequate brain circuits, as far as the inventiveness in generating alternative reappraisals is concerned. This inventiveness, however, is a necessary, but not a sufficient prerequisite for effective emotion regulation, since individuals not only need to be theoretically capable of generating suitable reappraisals for critical situations, they also need to do so when faced with these situations in daily life. Here, how men and women actually make use of their capabilities might critically depend on gender roles, which likely entail different beliefs in emotion regulation self-efficacy, reappraisal effectiveness, or controllability of stressors. However, these notions remain speculative until further investigation.

This study presents a novel approach for investigating gender differences in cognitive reappraisal by explicitly testing performance in generating alternative cognitive re-interpretations for anxiety-evoking situations. By drawing on an actual behavioral performance measure instead of self-reported data, our measure of reappraisal capacity is independent from the participants’ ability or willingness to accurately report on their abilities. *Post hoc* power analysis confirmed that at 0.989, our results are unlikely to be skewed by a type 2 error for women. Some limitations of this study must be noted. Naturally, the capacity to generate multiple cognitive reappraisals as assessed in this study only covers a certain aspect of an individual’s ability to effectively implement cognitive reappraisal for negative affect regulation. While specifically for situations that exceed routines, it can assumed that the likelihood for effective reappraisal implementation increases with the pool of generated ideas, for recurrent negative events in daily life, the ability to repeatedly implement just one reappraisal in a successful manner may be equally or even more important. Yet, since recurrent anxiety-eliciting situations (e.g., walking home alone at night) are not always exactly alike, a high capacity to generate manifold reappraisals may still prove vital. Secondly, it may be questioned why depression and not anxiety was used as an outcome variable when testing gender-specific effects of cognitive reappraisal capacity for anxiety-eliciting situations. Depression and anxiety greatly overlap; they share a great proportion of their symptomatology, as well as common genetic and environmental contributors (e.g., Preisig et al., 2001; Kessler et al., 2005; Burton et al., 2015). Yet, compared to anxiety, markedly more literature indicated correlations between depression and emotion regulation strategies, particularly cognitive reappraisal (e.g., Martin and Dahlen, 2005; Aldao et al., 2010; Joormann and Gotlib, 2010; Troy et al., 2010; Everaert et al., 2017). Thirdly, our claim that men and women possess similar cognitive reappraisal capacity and related executive functioning is based on experimentally instructed reappraisal within a limited time span. That is not to say that gender differences might not emerge when reappraisal time increases, perhaps as a function of cognitive effort, as was proposed by others (McRae et al., 2008; Domes et al., 2010). Thus, more fine-grained investigations into gender differences at specific stages of the cognitive reappraisal process are warranted that go beyond the presumably very early stage of generating multiple potential reappraisals scrutinized in this study (e.g., selection of a suitable reappraisal, implementation of that reappraisal, etc.). In this respect, scrutinizing the time-course of cognitive reappraisal by means of EEG may be particularly informative as regards (neural) efficacy of the reappraisal process in men and women. Next, this study’s results are based on cross-sectional data, which do not allow causal interpretations of the relations. While the research background denotes cognitive reappraisal capacity as the cause and depressive experiences as the effect (e.g., Hofmann et al., 2012; Berking et al., 2014), circular mechanisms may also be at work. In this respect, other studies suggested that deficits in implementing effective emotion regulation strategies might also arise as a consequence of depressive episodes (e.g., Troy et al., 2010; Liu and Thompson, 2017). Additionally, sex hormones and phases in menstrual cycle are known to affect emotional responding, including emotion regulation strategy choice (Toffoletto et al., 2014; Graham et al., 2018). Although in the present study, women with and without use of hormonal contraceptives did not differ in any variables of interest, we did not control for menstrual cycle data in our analyses, which constitutes an important direction in future research. Moreover, although we attempted for a comprehensive interpretation of our findings based on available literature, our propositions regarding potential influences of other variables on the relationship of reappraisal capacity and depressive symptoms (e.g., regulation effort, impact of other strategies) should be considered as preliminary until further studies demonstrate they significantly moderate the discussed effect. Also, note that our findings are restricted to reappraisal capacity in dealing with anxiety-eliciting events only. While reappraisal inventiveness can be regarded a trans-emotional capacity that is not specific to certain emotions (de Assuncao et al., 2015), gender differences might nonetheless emerge for the downregulation of anger, disgust, or sadness. Thus, a vital goal for future research is to identify whether the relationships identified in this study also hold for other versions of the RIT (e.g., anger, Weber et al., 2014). Lastly, this study used a sample of young students without severe mental health problems. Findings may not generalize to more serious depressive symptoms.

Taken together, the present study demonstrated that while men and women do not differ in their basic cognitive capacity to implement cognitive reappraisals in threatening situations, higher reappraisal capacity seemingly reduces depressive daily-life experiences in men only. This possibly implies a more complex link between cognitive reappraisal and depressive experiences in women, suggesting their benefits for well-being more strongly depend on several aspects of their emotion regulation efforts through reappraisal and beyond working in concert. Though preliminary, these findings may have useful implications for psychotherapy research and practice. For instance, whereas men might benefit from ability-based reappraisal trainings alone, in women, it may also need concomitant interventions that focus on reducing the use of maladaptive emotion regulation strategies as well as enhancing self-efficacy and determinedness in the context of cognitive reappraisal.

## Data Availability

The raw data supporting the conclusions of this manuscript will be made available by the authors, without undue reservation, to any qualified researcher.

## Ethics Statement

This study was carried out in accordance with the recommendations of the guidelines by the ethics committee of the University of Graz, Austria with written informed consent from all subjects. All subjects gave written informed consent in accordance with the Declaration of Helsinki. The protocol was approved by the ethics committee of the University of Graz, Austria.

## Author Contributions

EW, IP, and AF conceptualized the study. CP, IP, and CR collected, analyzed, and interpreted the data. CP drafted the manuscript. EW, IP, CR, AF, and HW critically reviewed the manuscript. All authors gave their final approval of the manuscript.

## Conflict of Interest Statement

The authors declare that the research was conducted in the absence of any commercial or financial relationships that could be construed as a potential conflict of interest.

## Footnotes

1 We adopted the current definitions of sex and gender, according to which sex is considered a biological component, which is defined via the genetic complement of chromosomes, whereas gender refers to the social, environmental, cultural, and behavioral factors and choices that influence a person’s self-identity and health (Clayton and Tannenbaum, 2016; National Institute of Health Office of Research on Women’s Health, 2019). Since it cannot be determined that any of the effects discussed in this study are caused by biological factors alone, differences between men and women are referred to as “gender differences." This does, however, not exclude the possibility that biological and social factors may interact in explaining the present results. If cited literature addressed sex or gender differences, their wording was adopted.
