# Supplementary material for: Gender Differences in Generating Cognitive Reappraisals for Threatening Situations: Reappraisal Capacity Shields Against Depressive Symptoms in Men, but Not Women
Source: Front Psychol. 2019 Mar 15;10:553. doi: 10.3389/fpsyg.2019.00553 (PMC6428936; doi:10.3389/fpsyg.2019.00553)
Supplement: Supplementary file 1 [file Table_1.DOCX]

**Appendix: Examples of reappraisal ideas coded into the four reappraisal categories**

(Weber et al., 2014; Assuncao et al., 2015).

Situation: *“At night, you lie alone in bed and are about to fall asleep, when you suddenly hear a loud noise from the living room. You get up, go into the living room and realize that the window is open”*

1. Problem-oriented reappraisal

„I will go to the window and figure out what happened“

“I will turn on the light and see for myself that no one is there”

“I will just look the door to my room, and then nobody can come in”

1. Positive re-interpretation

„I am stronger than any burglar, I can overpower anyone with ease“

“Great, now that I am awake, I get to treat myself to a hot cocoa”

“Now that the window is open, I can see how beautiful the night is”

1. De-emphasizing

„That was just the wind, nothing to worry about“

“Perhaps I just forgot to close the window properly before going to bed”

“Nothing bad can happen; I live on the third floor”

1. Symptom re-interpretation

„This is just my body overreacting, why should I be scared?“

“I will just keep calm and get my heart beat under control”
